# Acute brain injury and nanomedicine: sex as a biological variable

**DOI:** 10.3389/fbiom.2024.1348165

**Published:** 2024-02-02

**Authors:** Amberlyn Simmons, Olivia Mihalek, Heather A. Bimonte Nelson, Rachael W. Sirianni, Sarah E. Stabenfeldt

**Affiliations:** 1 School of Biological and Health Systems Engineering, Arizona State University, Tempe, AZ, United States,; 2 Department of Neurological Surgery, UMass Chan Medical School, Worcester, MA, United States,; 3 Department of Psychology, Arizona State University, Phoenix, AZ, United States

**Keywords:** sex-related, nanomedicine, nanoparticle, traumatic brain injury, stroke, sex differences

## Abstract

Sex as a biological variable has been recognized for decades to be a critical aspect of the drug development process, as differences in drug pharmacology and toxicity in female *versus* male subjects can drive the success or failure of new therapeutics. These concepts in development of traditional drug systems have only recently begun to be applied for advancing nanomedicine systems that are designed for drug delivery or imaging in the central nervous system (CNS). This review provides a comprehensive overview of the current state of two fields of research - nanomedicine and acute brain injury—centering on sex as a biological variable. We highlight areas of each field that provide foundational understanding of sex as a biological variable in nanomedicine, brain development, immune response, and pathophysiology of traumatic brain injury and stroke. We describe current knowledge on female *versus* male physiology as well as a growing number of empirical reports that directly address sex as a biological variable in these contexts. In sum, the data make clear two key observations. First, the manner in which sex affects nanomedicine distribution, toxicity, or efficacy is important, complex, and depends on the specific nanoparticle system under considerations; second, although field knowledge is accumulating to enable us to understand sex as a biological variable in the fields of nanomedicine and acute brain injury, there are critical gaps in knowledge that will need to be addressed. We anticipate that understanding sex as a biological variable in the development of nanomedicine systems to treat acute CNS injury will be an important determinant of their success.

## 1 Introduction

The consideration of sex as a biological variable was mandated by the National Institutes of Health (NIH) in 2015 (National Institutes of Health, 2015), recognizing that health and disease processes can vary across the sexes. These differences likely impact individual variations in diagnostics, etiology, prevention, and response to health, disease, and associated therapeutic treatments. Molecular differences between the sexes are increasingly recognized to hold implications for regulation of drug pathways, with evidence that there are marked sex and gender differences in drug efficacy and adverse events; yet, even as of 2020, the majority of drugs lack sex-dependent dosing recommendations (Zucker and Prendergast, 2020; Karlsson Lind et al., 2023). Thus, although evidence is accumulating that sex is a recognized variable influencing efficacy and outcome of traditional drug therapies, much remains unknown about how biological sex impacts development of pharmacotherapies, including the important aspect of nanoparticle systems. We focus our discussion primarily on nanoparticles, defined here as sub-micron colloidal systems. This definition includes synthetic or naturally derived particles with diameter less than 1,000 nm that are suspended within a fluid medium. Biologics, cells, antibody-drug conjugates, and injectable biomaterials are excluded from our analysis.

Several recent reviews have covered this topic from a broad perspective (Serpooshan et al., 2018; Hajipour et al., 2021; Sharifi et al., 2021; Poley et al., 2022). Here, we will focus on highlighting sex differences in both nanomedicine and acute brain injury whereby the intersection of these two areas impacts therapeutic development. We note that for the purposes of this review “sex” refers to the biological construct, rather than the social construct of “gender.” Our review is structured to address the foundations of sex differences, central nervous system (CNS) barriers in health and disease, and the interplay between the CNS and the immune system. From this foundation, we will then describe what is known in the field regarding sex differences in nanoparticle delivery to the CNS, focusing on specific examples in acute brain injury. Finally, we will highlight key observations and gaps in the field which should motivate future work.

## 2 Absorption, distribution, metabolism, and excretion (ADME)

Biological sex is a factor that is well-understood to influence the absorption, distribution, metabolism, and excretion (ADME) of a variety of bioactive agents, including small molecules, protein therapeutics, and oligonucleotides (Farkouh et al., 2020). These differences can be highly clinically significant and are increasingly essential considerations in the drug development process (Tannenbaum et al., 2019). For example, between 1997 and 2000, 8 of the 10 drug withdrawals that occurred in the US market occurred due to greater risks of adverse events in women compared to men (Soldin and Mattison, 2009). Sex differences can originate as a function of behavior (for example, differences in dietary or activity patterns, injury or disease context, likelihood of seeking care, clinician responsiveness, or patient compliance), or they may be a result of underlying biological differences between females and males (for example, differences in organ or tissue physiology, cellular behaviors, gene expression, or organizational and activational effects of gonadal hormones). The interplay between sex, behavior, and biology is often complex. In one example, female patients undergoing treatment for hypertension were observed to be less compliant than male patients, suggesting that behavioral interventions were merited to optimize therapy; however, accumulating evidence from deeper analyses suggests that innate biological differences in response to this specific drug therapy drove differential responses to therapy as well (Consolazio et al., 2022). The impact of failing to consider sex differences in ADME is multifaceted, potentially involving unexpected toxicity or poorly optimized dosing paradigms, which leads to insufficient efficacy as well as putatively unnecessary adverse events (Madla et al., 2021). Thus, sex is an increasingly important factor understood to influence ADME to drive therapeutic development considerations. Here, we will overview the major principles of ADME and consider how these concepts may be extended for development of sex-optimized nanomedicines.

### 2.1 Physiological basis for sex differences in ADME

**FIGURE 1:**
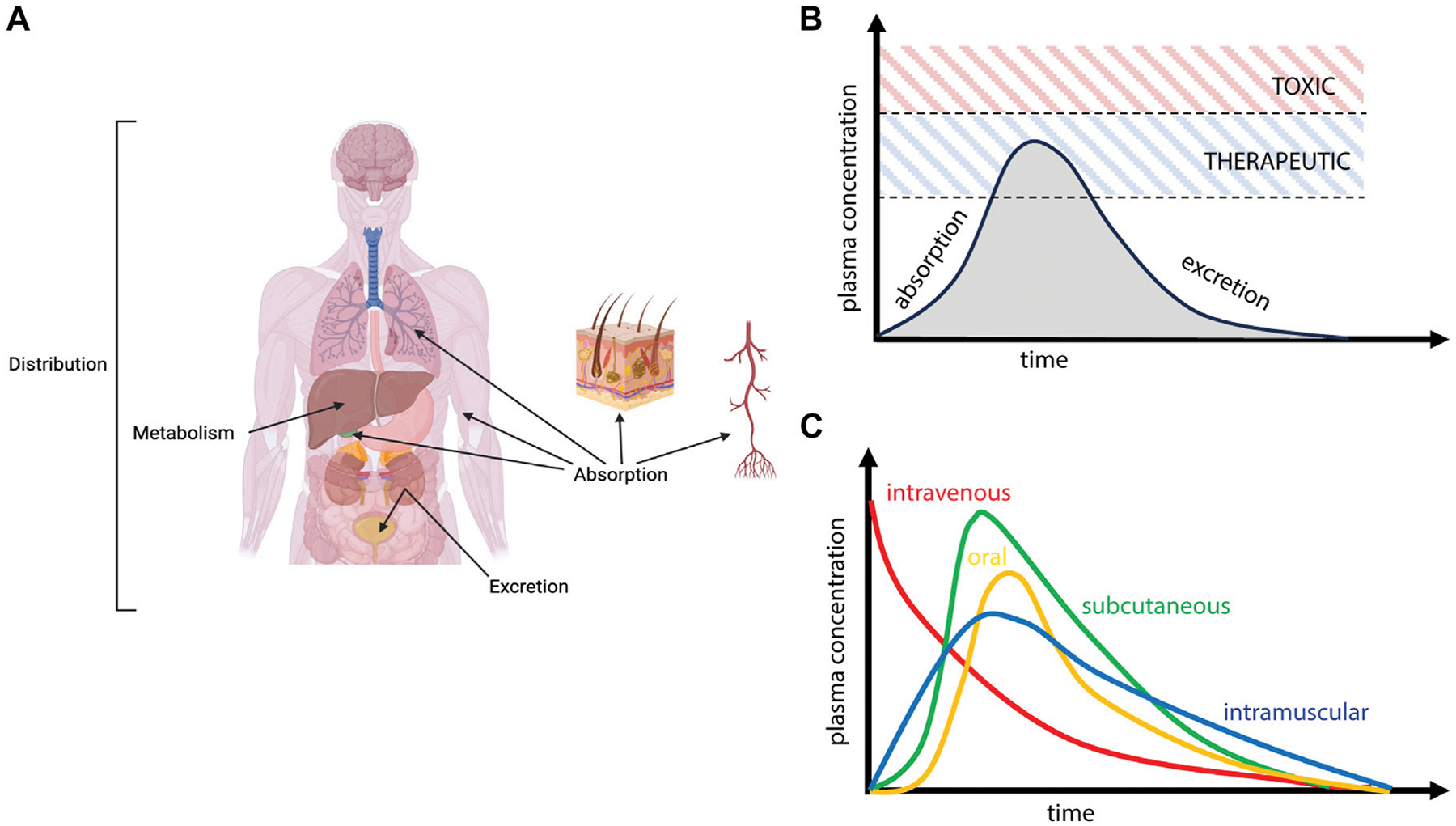
Schematic overview of Absorption, Deposition, Metabolism, and Excretion (ADME) processes. **(A)** Following administration, drugs enter the vascular compartment (absorption), distribute throughout various organs and tissues (deposition), experience biotransformation (metabolism), and ultimately clear from the body (excretion). **(B)** Circulating levels of drug reflect these competing processes of ADME, with absorption primarily defining the initial rise in plasma concentration and various deposition, metabolism, and excretion processes (elimination) defining the curve’s decay. Achieving therapeutic levels of drug while avoiding toxicity is the primary driver of drug efficacy. **(C)** The time course of plasma pharmacokinetics will depend heavily on route of administration.

ADME refers to the process by which a molecule enters, moves through, and is ultimately cleared from the body (Figure 1). Absorption is the process by which an administered compound reaches the bloodstream. For drugs that are dosed intravenously (IV), agents will rapidly partition within the vascular compartment, which is a heterogeneous medium composed of fluid, proteins, lipids, platelets, and cells; sex-dependent interaction of drugs with these components is known to impact clinical pharmacokinetics and subsequent toxicity or efficacy (Routledge et al., 1981). For drugs that are dosed orally, absorption is initiated in the gastrointestinal tract, where sex differences in pH, enzyme secretion, gastric volume, and gut transit time have been shown to alter the uptake of bioactive agents into circulation (Stillhart et al., 2020). The microbiome also exhibits significant and well-studied sex differences, which have been proposed to impact the gut-brain axis leading to sex differences in disease phenotype and drug ADME (Jašarević et al., 2016; Hokanson et al., 2023). For intranasal dosing, drugs first encounter the nasal passageways and mucosal barriers, for which mucous pH, secretion, and clearance have been shown to be sex-dependent (England et al., 1999; Hallschmid, 2021; Luberti et al., 2021; Marjan, 2023). Additional sex differences have been observed for drug ADME through ocular (Nakamura et al., 2005), intravascular (Kunio et al., 2018), and subcutaneous (Sloan, 2000) dosing paradigms; physiological considerations for these parenteral routes of administration are reviewed elsewhere (Donovan, 2005).

**FIGURE 2:**
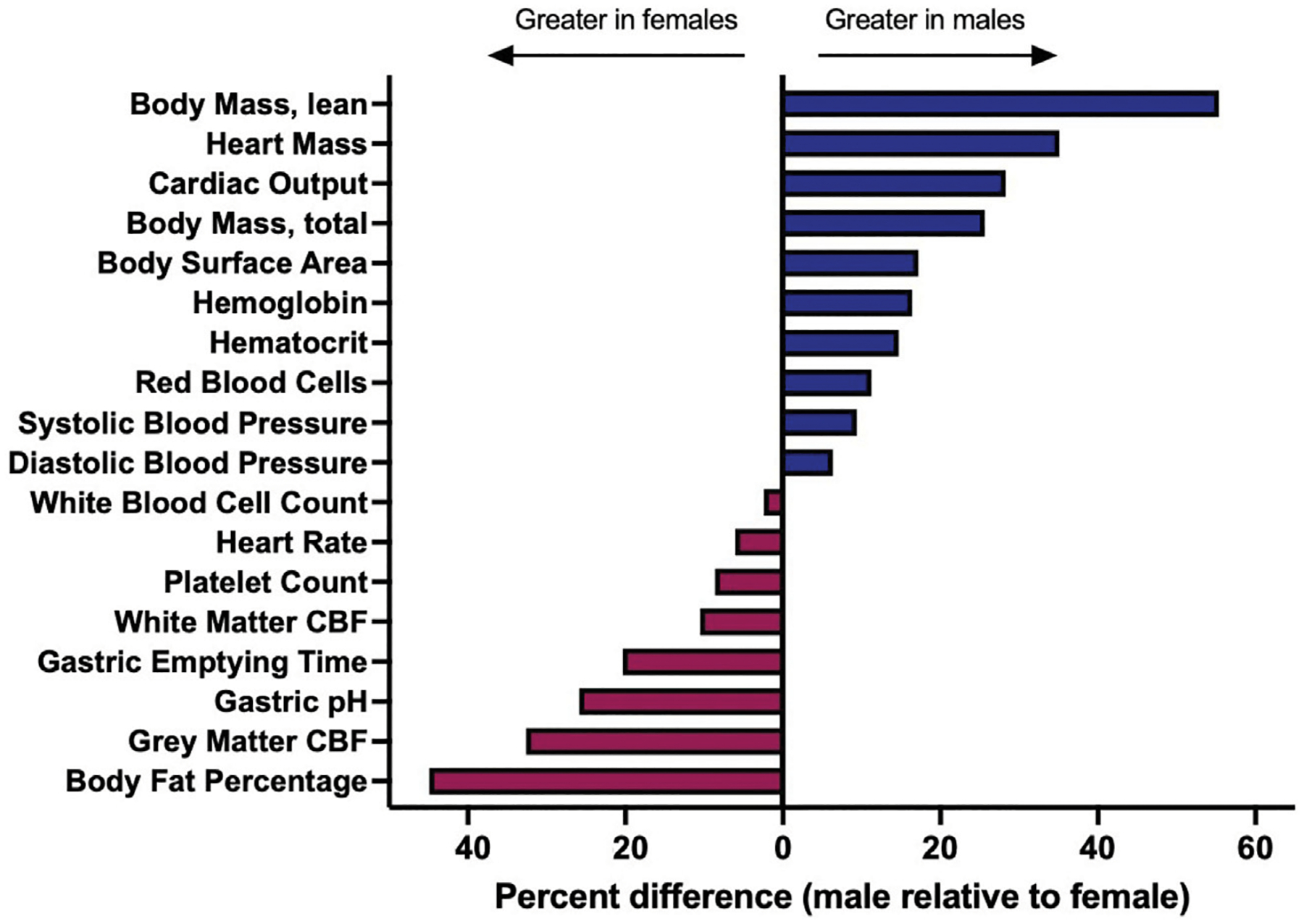
A variety of underlying physiological parameters depend on sex. Physiological parameters are described for healthy, adult populations. Percent difference was calculated by the following formula: (male-female)/female. Sources and raw data for each value are provided in Supplementary Material. Abbreviations: cerebral blood flow [CBF].

Once a drug has been absorbed into systemic circulation, it will face similar physiological barriers within the vascular compartment, and it is from this source that therapeutic agents will distribute to peripheral tissues as well as the CNS. Drug distribution may depend on a multitude of factors that are sex-dependent, including the physicochemical features of the drug governing its interaction with blood components, which limits or enhances bioavailability and passive movement into tissue, or indirect impacts on physiological processes that subsequently alter distribution on the basis of sex (Figure 2). In addition to the sex-differences in the binding of drugs to plasma proteins that have been noted in the field (Soldin and Mattison, 2009), there are well appreciated sex differences in blood volume, chemistry, and physiology, as well as for organ-specific vascular features (Boese et al., 2017). Males typically have higher blood volume and cardiac output, as well as lower rates of immune activation, compared to females. Males also possess lower quantities and distinct patterns of fat deposition on the body compared to females, which yields varying distribution patterns in female *versus* male subjects (Blaak, 2001; Karastergiou et al., 2012). These differences extend to other organ systems; for example, males also have a higher glomerular filtration rate in the kidneys (McDonough et al., 2023), exhibit profound differences in lung physiology (Townsend et al., 2012), and demonstrate well-appreciated metabolic and physiological differences in the liver (Maggi, 2022). With respect to CNS tissues, there are known differences in blood-brain barrier (BBB) integrity and physiology as a function of sex that would be expected to influence nanomedicine delivery. This includes established differences in shear stress responses from brain endothelial cells as well as more subtle possible alterations to transendothelial electrical resistance (TEER) in *in vitro* models and drug-specific metabolism at the BBB (Weber and Clyne, 2021). Sex differences that are specific to the central nervous system will be discussed in greater detail in the sections that follow.

Once within a physiological environment, all therapeutic agents are subject to metabolism, a process or series of processes that can either enhance or inhibit activity of the compound. A significant portion of drug metabolism is driven by enzymes that are expressed in the liver and involve the cytochrome P450 (CYP) family of enzymes, although metabolism is a complex process that can occur in and involve multiple organ systems and enzyme classes. Differences in enzyme expression play a critical role in determining drug clearance from circulation, degradation within tissue compartments, and subsequent toxicity of systemically administered therapeutics. Metabolic processes are well-understood to be sex-dependent and are a primary consideration in therapeutic development for traditional drugs (Waxman and Holloway, 2009). In the case of nanoparticle-based drug delivery systems, nanoparticle distribution to organ systems is primarily driven by the properties of the nanoparticles; drug metabolism itself is outside of the scope of this review, and so we direct the reader to other abundant resources on this topic (Valodara and Johar, 2019).

Excretion is a key process governing the fate of both drugs and nanoparticles in the body, with the liver and kidneys being primary routes through which substances exit the body. Nanoparticles also clear from circulation via the reticuloendothelial/mononuclear phagocytic systems (RES and MPS, respectively), which ultimately leads to clearance and/or deposition of nanoparticle systems in lymph nodes and spleen (Tang et al., 2019). Some evidence suggests that the MPS exhibits sex differences (Varghese et al., 2022), although other works suggest there is not a sex difference (Wang et al., 2021). This mechanism of elimination is particularly important for CNS delivery, considering that rapid depletion of vascular concentration will all but prevent CNS delivery. Engineering nanoparticles to avoid the phagocytosis by circulating immune cells is thus an approach of focused interest in nanoparticle development (Wen et al., 2023).

## 3 Sex differences in the brain

It is evident that biological sex influences all aspects of drug delivery, including how the drug interacts with the body and how the body reacts to the drug. Keeping these critical sex differences in ADME in mind, we now highlight sex differences in the brain and discuss how these differences may impact nanomedicine delivery to the brain in the context of acute injury.

### 3.1 Sex determination and its effects on the brain

The phenotypic expression of sex differences is due to the effects of gonadal hormones at organizational (permanent) and activational (transient) levels, as well as the impact of sex chromosomes (Koebele and Bimonte-Nelson, 2015; Arnold, 2022). Recent advancements have led to the understanding that there are gene agents on both the X and Y chromosomes that are related to mammalian sexual differentiation, in turn driving sex differences in circulating hormone levels yielding sex differences in the brain. In early embryogenesis, the Sex-determining Region Y (*Sry*) gene on the Y chromosome, also known as the testis-determining gene, directs the bipotential gonadal ridge to differentiate into testes and inhibits ovarian development (Cabrera Zapata et al., 2022). Ovarian differentiation in XX individuals is due in part to X-linked genes, although the specific identification of these agents and their resultant functions are as-yet unclear (Arnold, 2022; Cabrera Zapata et al., 2022); deciphering these factors are critical for future directions of research. Sex chromosomes drive gonadal hormone expression, and while detailed discussion of hormonal impacts and trajectories on sex differences in the brain is beyond the scope of this review, it is important to note that gonadal steroids, including estrogens, progesterone, testosterone, dihydrotestosterone, and androstenedione, are among the key players that drive these differences (for further review and discussion see: (Koebele and Bimonte-Nelson, 2015; 2017). Notably, effects of these hormones on the brain and its functions, during development and beyond into old age, are both individual and interactive with organizational sex hormone actions setting up tissues to “activate” in a particular fashion when exposed to sex hormones at a later time.

### 3.2 Cerebrovasculature function

Sex differences have been observed for cardiac, vascular, and cerebrovascular physiology, and these differences could impact delivery of either drugs or nanomedicine to the CNS. Considering the cardiovascular system, females typically have smaller diameter blood vessels, reduced total blood volume, a higher heart rate, lower blood pressure, and a net lower cardiac output than males (Zaid et al., 2023). Recent literature has highlighted that differences in fluid physiology may yield distinct shear stress and vascular reactivity in females *versus* males (Pabbidi et al., 2018; James and Allen, 2021), which could be a particularly important factor influencing the interaction of circulating colloids with brain endothelial cells. Females have previously been reported to have higher cerebral blood flow compared to males, which may be largely affected by sex steroid hormones (Robison et al., 2019). Expression of sex steroid hormone receptors are established in cerebral microvasculature. Estradiol binding to its receptor on endothelial cells has previously been implicated in the maintenance of the vascular system (Cho et al., 1999; Lösel and Wehling, 2003; SenthilKumar et al., 2023). For example, *in vitro* studies have revealed estradiol binding to endothelial cell receptors activates endothelial nitric oxide synthase, which leads to elevated nitric oxide levels and changes in paracellular permeability (Cho et al., 1999). Additionally, the role of androgens in angiogenesis, reactivity, integrity, and inflammation have previously been described (Abi-Ghanem et al., 2020). For instance, Atallah and others show chronic testosterone depletion via castration of 8-week-old male mice leads to increased cerebrovascular permeability, which was evident by multiple tracers and decreased expression of proteins that provide structural integrity (Atallah et al., 2017). They also show testosterone depletion induces increased expression of pro-inflammatory cytokines (IL-1B, TNF-a) and glial cell activation markers (IBA-1, GFAP), indicating increased inflammatory responses. Notably, they found testosterone supplementation after 5 weeks restores cerebrovascular integrity and results in no significant differences in inflammatory state between castrated and intact males. Furthermore, multiple lines of evidence support sex differences in cardiovascular health and sex-dependent risk of cardiovascular disease (CVD), which can be attributed to steroid hormone profiles (SenthilKumar et al., 2023). For example, prior to menopause, females exhibit a lower risk for developing CVD compared to males. However, alterations in sex-steriod hormone profiles associated with menopause lead to increased risk of CVD in females as compared to males.

### 3.3 Blood-brain barrier (BBB)

#### 3.3.1 Healthy BBB physiology

The BBB is composed of brain endothelial cells that create a continuous, non-fenestrated microvascular network in the central nervous system (CNS) (Cho et al., 1999). A detailed review of the structure and function of the BBB was recently given by Zhang et al. (Zhang et al., 2022). In short, tight junctions, adherens junctions, and gap junctions between endothelial cells control the movement of ions, molecules, and cells from the blood to the brain parenchyma (Hawkins et al., 2006). Tight junctions are created by homotypic and heterotypic interactions between transmembrane proteins including claudins, occludins, and junction adhesion molecules (JAMs), that connect to the actin cytoskeleton via cytoplasmic scaffold proteins of the membrane-associated guanylate kinase (MAGUK) family (Yuan et al., 2020; Hashimoto et al., 2023; Sugiyama et al., 2023). Conversely, adherens junctions are formed by members of the cadherin protein family and are linked to the actin cytoskeleton by catenins (Weis and Nelson, 2006). A thin sheet of basement membrane (BM) composed of glycosaminoglycans, proteoglycans, and glycoproteins surrounds the endothelial barrier and provides structural support to the neurovascular network (Tewari et al., 2022). The composition of the brain extracellular matrix (ECM) changes with age and disease, which has effects on the integrity of the BBB (Thomsen et al., 2017); ECM composition as well as biomechanical properties have been reported to vary by sex (Batzdorf et al., 2022). Surrounding the BM are pericytes, astrocytes, and microglia that secrete ECM molecules and other signals to influence the BBB (Bisht et al., 2021; Kisler et a., 2021; Gao et al., 2023). Together, these glial cells offer homeostasis and structural support to the BBB, and any deviation can result in disease (Tewari et al., 2022). Pericytes reside within the BM and communicate with endothelial cells and astrocytes through the secretion of signals (Armulik et al., 2010; Wu et al., 2023). Astrocytes extend end foot processes into the BM, where they play major roles in the clearance of waste, regulating BBB permeability, and modulating synaptic transmission (Abbott et al., 2006; Tewari et al., 2022; Wu et al., 2023). Microglia are the brain’s resident immune cells that are responsible for innate and adaptive immune responses. These glial cells, along with the endothelium and local neurons, are termed the neurovascular unit (Zlokovic, 2008; Sato et al., 2022).

Sex-related differences in the behavior of glial cells in non-disease states have previously been reported. An in-depth review conducted by Lenz and McCarthy describes the various roles microglia play in brain development, including regulation of neural stem cell populations, synaptogenesis, and sexual determination (Lenz and McCarthy, 2015). Microglia in multiple regions of the developing rodent brain show sex differences in colonization, gene expression, and cytokine secretion (Schwarz et al., 2012). Sex differences in microglia phenotypes also extend into adulthood in animal models and are retained independently from the *in vivo* steroid hormone profile (Villa et al., 2018; Doran et al., 2019). These observed differences in phenotype establish that male and female microglia behave distinctly. Additional studies are needed to determine the extent that sex-related differences in microglia phenotypes impact the BBB. Similarly, sex steroid hormones influence astrocyte function and morphology. Studies have shown that estradiol is implicated in the expansion of astrocyte processes and regulation of intracellular ion concentration (Acaz-Fonseca et al., 2014). Importantly, this may have profound sex-dependent effects on the communication between astrocytes and other cells of the BBB, which is yet to be explored. Further research is required to elucidate sex-dependent function of the neurovascular unit under normal physiological conditions.

#### 3.3.2 Injured BBB physiology

The permeability of the BBB is tightly regulated by glial cells, which work together to sense the environment and produce signals to alter the flow of materials from the blood to the brain tissue. In the context of disease or injury, the normal signaling between glial cells and the brain endothelium is disrupted, resulting in a dysfunctional BBB (Xing et al., 2012). Microglia become activated when surface receptors come in contact with damage-associated molecular patterns (DAMPs) or pathogen-associated molecular patterns (PAMPs), which leads to the activation of pro-inflammatory machinery and induced activation of surrounding microglia (Lawrence et al., 2023). Activated microglia phagocytose cellular debris caused by the injury or disease, leading to the release of signals such as tumor necrosis factor-α (TNF-α) that act on local astrocytes. The subsequent change in gene expression and phenotype of astrocytes in response to the local environment is termed reactive astrogliosis. Reactive astrocytes promote pro-inflammatory conditions by secreting complement components and chemokines that attract circulating peripheral immune cells (Liddelow and Barres, 2017). These disease processes exacerbate oxidative stress and BBB dysfunction through the generation of pro-inflammatory signals, free radicals, and matrix metalloproteinases (MMPs) (Zhao et al., 2022). Secretion of pro-inflammatory cytokines, such as IL-1B and TNF-α, degrade tight junction proteins via multiple signaling pathways. Overproduction of free radicals by microglia and astrocytes, along with any inherent disease-induced free radical accumulation, leads to cellular damage and further disruption of the BBB. In parallel, the secretion of MMPs intensifies BBB breakdown through the direct digestion of tight junction and adherens junction proteins. In summary, the pathophysiology of the injured BBB is complex and involves all glial subtypes. Adding to this complexity, previous literature has illuminated sex-related and hormone-related differences in glial cell response to pathological conditions. A recent study of female primary cortical astrocytes reveals estradiol and 5ɑ-androstane-3β,17β-diol (a dihydrotestosterone metabolite) may offer protection against cytotoxic challenge with iodoacetic acid (Kim et al., 2023, p. 3). The authors speculate that the protective effects may result from steroid induced inhibition of connexin 43 gap junction opening. Specifically, this channel is important for cellular signaling and changes in channel permeability have been associated with disease states. Estradiol has also previously been implicated in pathways involved in pericyte attachment and migration in response to inflammatory stimuli (Kurmann et al., 2021). Kurmann and others show estradiol treatment prevents cultured human brain vascular pericyte migration through estrogen receptors ER-ɑ and ER-β signaling. Furthermore, their transcriptomics analysis reveals that estradiol treatment induces changes in expression of transcripts associated with cell migration under inflammatory conditions. Since astrocytes and pericytes are involved in maintaining BBB integrity, these findings suggest a critical role of hormone-mediated protection in the context of brain injury. Additionally, previous studies reveal *ex vivo* cultures of male neonatal rat microglia stimulated with lipopolysaccharide (LPS) have increased pro-inflammatory responses compared to female cultures (Loram et al., 2012). This leads into the next section, which discusses how biological sex plays a role in peripheral immune responses to brain injury.

## 4 Peripheral immune response to acute brain injury

Acute brain injury leads to peripheral immune responses, which are initiated by the infiltration of peripheral immune cells into the parenchyma and transport of DAMPs and PAMPs to draining lymph nodes. Here, we review the process of peripheral immune activation, how biological sex influences immune responses and emerging sex differences in innate immune cells involved in CNS injury.

### 4.1 Peripheral immune cell signaling

The important players in peripheral immune responses originate from multipotent hematopoietic stem cells that undergo differentiation to become common myeloid progenitor or common lymphoid progenitor cells (Kondo et al., 1997; Akashi et al., 2000). Common myeloid progenitors give rise to innate immune cells, including granulocytes, monocytes, macrophages, and dendritic cells. Conversely, common lymphoid progenitors differentiate into adaptive immune cells including T cells, B cells, and NK cells. These peripheral immune cells use signals from other cells as a road map to reach the brain when injury or disease occurs. An in-depth review conducted by Besedovsky and Rey discussed how neural mediators, such as neurotransmitters, act on immune cells to induce a variety of responses (Besedovsky and Rey, 1996). They also examined the effects that immune-derived products have on the cells of the CNS, including neurons and astrocytes. Another important group of signals that are involved in this neuro-immune axis are chemokines, which are small proteins that initiate peripheral immune cell migration and maturation (Rostène et al., 2007). Together, neural mediators, immune-derived products, and chemokines provide a means of communication for these systems that aids in maintaining homeostasis, preventing disease, and healing injury.

### 4.2 Immune activation through dura and glymphatic system

The brain was once considered an immune privileged organ, however, more recent studies highlight the presence of resident immune cells within various compartments of the CNS (Buckley and McGavern, 2022; González-Hernández and Mukouyama, 2023). One major site for CNS immune surveillance is the dura mater, a collagenous membrane forming the outermost layer of the meninges. The dura mater contains fenestrated blood vessels, allowing for the trafficking of peripheral immune cells and other materials. Under inflammatory conditions, peripheral immune cells travel from the dura to the meninges via chemokines, which facilitates their access to the parenchyma. Importantly, the newly named glymphatic system provides means of waste removal and small molecule exchange between cerebral spinal fluid (CSF) and interstitial fluid (ISF) that ultimately drains to peripheral lymphatics (Iliff et al., 2012). Astrocytic aquaporin-4 (AQP4) channels enable this movement of CSF into the parenchyma, as well as removal of ISF from the parenchyma. Analogous to the peripheral lymphatic system, the glymphatic system provides means of travel for DAMPs from the CNS to peripheral immune organs such as the spleen and lymph nodes. This results in the activation of peripheral immune cells that initiate systemic immune responses.

### 4.3 Sex differences influence immune responses

Differences in sex steroid hormone profiles and genetics play roles in sex-specific immune responses. Innate and adaptive immune cells express a variety of hormone receptors including estrogen, progesterone, and androgen receptors. Sex hormones have a variety of implications in immune cell development and function. Generally, estrogens have been reported to promote enhanced humoral immunity, while androgens promote immunosuppression and immunomodulatory effects (Sciarra et al., 2023). Thus, differences in sex-steroid hormone profiles between males and females may have an impact on immune responses. Additionally, sex differences in immune responses have previously been linked to genetics. The human X chromosome encodes a variety of genes related to immune regulation including transcription factors, cytokine receptors, toll-like receptors (TLRs) (Klein and Flanagan, 2016). Some of these genes can escape X inactivation and show increased expression in females compared to males. In the next section we focus on emerging evidence of sex differences in innate immune cells and how it may impact acute brain injury.

### 4.4 Sex differences in innate inflammation

The innate immune system is rapidly activated and plays a crucial role in neuroinflammation after acute brain injury. Previous studies have revealed neutrophils and monocytes are two key innate immune cells involved in the response to brain injury (Jin et al., 2012). These cells play multiple roles in the immune response including debriding tissue, producing signaling molecules, and acting as a bridge between the innate (tissue resident) and adaptive (peripheral) immune systems (Mantovani et al., 2011; Hazeldine et al., 2014).

#### 4.4.1 Neutrophils

Neutrophils are one of the first peripheral immune cells to reach the CNS and may persist chronically after injury (Alam et al., 2020; Mohamud Yusuf et al., 2022). Despite their initial avail, neutrophils are implicated in pathogenic processes associated with chronic neuroinflammation, such as hyperpermeability of the BBB, edema, and reactive oxygen species (ROS) metabolism (Liu et al., 2018). Notably, recent studies have revealed sex differences in neutrophil activation, function, and apoptosis. A multi-omic analysis of mouse neutrophils revealed sex differences in chromatin architecture, with male cells exhibiting increased chromatin compaction compared to female cells (Lu et al., 2021). Chromatin architecture is an important aspect of neutrophil biology due to its involvement in neutrophil extracellular traps (NETs), and the observed sex differences in chromatin architecture may contribute to sex-specific neutrophil responses and gene expression. Another study of isolated human neutrophils revealed that cells obtained from male subjects exhibit higher expression of genes associated with immature phenotypes compared to female cells (Gupta et al., 2020). Neutrophils isolated from healthy females show increased activation, as evident by female-specific gene set enrichment of pathways related to type I interferon signaling and stimulation in the absence of stimulus. It was also noted that male neutrophils treated with estradiol show similar mitochondrial metabolism compared to untreated female neutrophils, which suggests that differences in steroid hormone profile, rather than X chromosome dosage, lead to sex-specific neutrophil phenotypes. To test this hypothesis, researchers analyzed neutrophil type I interferon signaling/stimulated genes in males with Klinefelter syndrome (XXY) and prepubescent volunteers. Female (XX) neutrophils consistently exhibited higher expression of type I interferon signaling/stimulated genes compared to XY and XXY males, and no significant statistical differences in expression between the XY males and XXY males. Additionally, there were no significant differences in the maturation profile or expression of type I interferon signaling/stimulated genes of prepubescent male and female neutrophils. Together these findings suggest that sex steroid hormones contribute to determining female-specific neutrophil physiology in healthy individuals. There are also reports of sex-specific neutrophil responses to pro-inflammatory signals. For example, Pace and others demonstrated that neutrophils isolated from human male subjects exhibit increased production of prostaglandin-E2 (PGE2) upon stimulation with lipopolysaccharide compared to those isolated from female subjects (Pace et al., 2017). PGE2 is a bioactive molecule involved in homeostatic and inflammatory processes. The authors hypothesize this may be due to sex differences observed in COX2 expression, as this is the key enzyme involved in prostaglandin biosynthesis. Ultimately, these findings show there are sex differences in neutrophil biology, and additional research is needed to understand the extent of these effects. Given that neutrophils play a crucial role in inflammation after brain injury, sex differences in neutrophil function may contribute to the sex differences in CNS pathology.

#### 4.4.2 Monocytes and macrophages

Monocytes, like neutrophils, infiltrate the CNS rapidly in the event of an injury in response to chemoattractants (Jin et al., 2012; Alam et al., 2020). Once monocytes have entered the brain tissue they mature into tissue resident macrophages and begin to perform effector functions including production of inflammatory mediators and phagocytosis of cellular debris (Yang et al., 2014; Alam et al., 2020). Recent studies have revealed hormone-related and sex-related differences in macrophage development and characteristics. Consiglio and others show androgen receptor signaling plays a role in monocyte development in male mice (Consiglio and Gollnick, 2020). They found that deletion of androgen receptors in myeloid cells leads to reduced numbers of mature monocytes and increased numbers of macrophages in bone marrow compared to controls, suggesting androgen receptor signaling increases monocyte development. Additionally, a study of isolated peritoneal macrophages from young and middle-aged rats revealed young females exhibit an increased percentage of macrophages that express activation markers toll-like receptor 4 (TLR4) and major histocompatibility complex II (MHCII) compared to young males (Ćuruvija et al., 2017). It was also reported that when stimulated with lipopolysaccharide (LPS), isolated macrophages from young female rats produced higher levels of interleukin 6 (IL-6) and interleukin 1β (IL-1β) compared to young male macrophages. These findings highlight the impact of sex and age on macrophage phenotypes and response to inflammatory stimuli in animal models. Future studies should focus on elucidating sex-related and hormone-related macrophage responses to better understand the roles they may play in CNS injury. The next section will review reported sex differences in traumatic brain injury (TBI) and stroke.

## 5 Sex differences in acute CNS injury

### 5.1 Traumatic brain injury (TBI)

#### 5.1.1 Epidemiology

As one of the leading causes of injury-related death and disability, TBI, affects between 50 and 60 million people globally each year (Maas et al., 2022). The global economic burden of the disease is estimated to be $400 billion per year. Previous literature reveals males have higher rates of TBI compared to women (Mikolic et al., 2021; Centers for Disease Control and Prevention, 2021; Centers for Disease Control and Prevention, 2022). In 2016 and 2017, males were reported to have higher rates of TBI-related hospitalizations for all injury mechanisms including unintentional falls, motor vehicle crashes, and assault (Centers for Disease Control and Prevention, 2021). Additionally, males were reported to have more than three times higher rates of TBI-related deaths compared to females in 2018 and 2019 (Centers for Disease Control and Prevention, 2022). Despite previous efforts to uncover sex differences in TBI outcomes in preclinical and clinical studies, this topic is still widely considered to be controversial. Remarkably, an extensive review by Gupte and others unveiled that human studies report females predominately experience poorer clinical outcomes than males following TBI (Gupte et al., 2019). They found that study parameters such as study size, stratification of TBI severity, and type of outcome measured have an impact on the extent of sex differences reported in human studies. Furthermore, similar trends extend to preclinical TBI models where the mechanism of injury, sample size, and type of outcome measured appear to have a considerable impact on reports of sex differences. Additional research in both preclinical and clinical domains is needed to decipher the etiology and magnitude of sex differences in TBI.

#### 5.1.2 Pathophysiology

TBI is characterized by a blow, jolt, or penetration to the head that results in the disruption of brain function, leading to complex pathological processes. The acute injury phase is a direct result of the initial injury, which includes damage to neurons, glia and blood vessels. Following the acute phase is a heterogenous, secondary injury phase that encompasses neuroinflammation, heightened intracranial pressure, dysfunction of the BBB, and neuronal excitotoxicity. Neuroinflammation begins with the activation of microglia, which secrete cytokines and chemokines to attract other immune cells such as neutrophils, monocytes, and lymphocytes, to the injury penumbra (Simon et al., 2017). While the initial inflammation aids in neuroregeneration and the clearance of cellular debris, chronic inflammation in the brain can lead to neuronal cell death and further neurodegeneration (Ziebell and Morganti-Kossmann, 2010; Simon et al., 2017). Transport of DAMPs to peripheral immune organs via the glymphatic system leads to systemic immune responses. For example, preclinical studies of TBI have revealed short-term increases in myeloid cell differentiation in the bone marrow and chronic changes in circulating immune cell profiles and behaviors (Ritzel et al., 2018). Additionally, examination of the thymus has revealed chronic disturbances in T cell maturation after TBI.

Recent preclinical studies have revealed sex differences in TBI pathology. Our group previously assessed macromolecule accumulation profiles in male and female mice after experimental TBI (Bharadwaj et al., 2020). Here, horseradish peroxidase (HRP) staining revealed females experience increased macromolecule accumulation in the injured cortex 24 h post-injury compared to males, suggesting sex-dependent BBB permeability after TBI. Additionally, Schwab and others found that female mice exhibit increased DNA damage compared to males after repetitive mild TBI (Schwab et al., 2022). Female mice exhibited increases in R-loops and oxidative base damage compared to sham levels, which was not evident when comparing sham males to injured males. However, when markers for cellular senescence were examined, males and females expressed comparable increases in p21 and p16 proteins compared to their sham counterparts. The authors stated this suggests that males might be more sensitive to genotoxic stress compared to females, and additional research is needed to elucidate the mechanisms that contribute to injury-induced sex-dependent DNA damage. Furthermore, Villapol and others also report that male mice show increased microglial activation, macrophage infiltration, and cell death compared to females at acute timepoints after moderate CCI (Villapol et al., 2017). In this study biological sex and time post-injury were reported to influence cytokine production in microglia and macrophages up to 30 days post-injury. For example, male mice exhibited increased TNF expression at 3 days post-injury compared to females, whereas females exhibited increased TNF and IL-1 expression at 4 h post-injury. Similarly, a study conducted by Doran and others also reported male mice exhibited increased myeloid cell infiltration at 1 day post-injury and increased microglia count at 3 days post-injury compared to females (Doran et al., 2019). Moreover, sex differences have been reported in clinical studies. Wagner and others evaluated sex differences in CSF glutamate concentration and lactate-pyruvate ratio after severe TBI in adults ages 16–65 years (Wagner et al., 2005). Here, females had higher lactate-pyruvate ratios, indicating females experience increased oxidative stress after severe TBI compared to males. They also report sex associations with 24-h glutamate concentration using multivariate analysis, indicating sex differences in glutamate excitotoxicity post-injury. Both findings provide evidence for sex differences after injury that directly affect cells of the BBB.

### 5.2 Stroke

#### 5.2.1 Epidemiology

Stroke affects approximately 12 million people worldwide each year (Feigin et al., 2021). Ischemic stroke (IS) accounted for about 60% of those cases and hemorrhagic stroke (HS) accounted for the remaining 40%. Notably, in 2019, stroke was the fifth leading cause of death for men and the third leading cause of death for women (Rexrode et al., 2022). Overall, women are considered to have a higher lifetime risk for stroke compared to men. However, age impacts the sex-related risk of stroke, with women having a higher incidence up to 30 years of age and men having a higher incidence thereafter until about age 80 (Vyas et al., 2021). There are also differences associated with the type of stroke experienced by men and women, with women having higher prevalence of subarachnoid hemorrhage and men having higher prevalence of hemorrhagic stroke (Rexrode et al., 2022). Previous studies have revealed sex-related differences in risk factors of stroke. For example, women diagnosed with diabetes have higher risk of stroke compared to men with diabetes (Peters et al., 2014). Some other factors that show sex associations include obesity, hypertension, atrial fibrillation (Vyas et al., 2021). Furthermore, there are female-specific risk factors for stroke including the use of hormone contraceptives and therapies, adverse pregnancy outcomes, and age of menopause onset. These phenomena may be due to sex and age-dependent alterations in circulating steroid hormone profiles and future investigations should lay emphasis on elucidating the causes of the sex-dependent risk of stroke.

#### 5.2.2 Pathophysiology

IS results from reduced blood flow that disturbs the normal function of the brain, causing neurological deficits. The most common causes of IS include arterial occlusion and venous infarction. Similar to IS, HS results in reduced blood flow to the brain caused by rupture of a cerebral artery rather than occlusion. Ruptures can occur in the cerebrum or in the subarachnoid space (SAS). In cases of HS, intracranial pressure rises quickly as the hemorrhagic blood moves into the SAS, ventricles, and parenchyma (Lauzier et al., 2023). At the core of the ischemic event and downstream from the ruptured vessel, the decrease in blood flow contributes to sustained ischemic conditions leading to neuronal death and secondary injury sequelae (Arai et al., 2011). Conversely, in the surrounding penumbra, other perfusing blood vessels provide some reprieve, resulting in a much slower process of cell death. However, in more severe cases, global ischemia can occur, which causes severe oxygen deprivation to large regions of the brain (Lauzier et al., 2023). In the absence of oxygen, neurons lose the ability to generate ATP and extracellular glutamate quickly accumulates causing excitotoxicity (Arai et al., 2011; Long et al., 2023). Additionally, mitochondrial damage leads to the release of free radicals causing oxidative stress and further damage to surrounding cells (Tian et al., 2022). Resident microglia play dual roles by producing pro-inflammatory cytokines and MMPs that cause further BBB and cellular damage, while also producing growth factors that aid in neuroprotection (Jayaraj et al., 2019). Studies of the consequences of stroke have revealed both acute and chronic systemic immune responses. The disruption of the blood brain barrier after stroke allows DAMPS and cytokines to enter the bloodstream, which leads to systemic immune responses. Increases in blood plasma levels of pro-inflammatory cytokines occur as early as 4 h after experimental ischemic stroke (Offner et al., 2006; Chapman et al., 2009). Assessment of splenocytes and circulating peripheral immune cells revealed increased secretion of pro-inflammatory cytokines in experimental models. Post-stroke assessments in humans have shown similar increases in peripheral pro-inflammatory cytokine (Ferrarese et al., 1999; Landreneau et al., 2018). Despite the initial systemic inflammatory response, the immune response shifts towards immunosuppression, termed stroke-induced immunosuppression (SIIS). As emerging key contributors to SIIS, immunosuppressive neutrophils and polymorphonuclear myeloid-derived suppressor cells (PMN-MDSCs) are proposed to induce systemic immunosuppression just days after stroke (Xie et al., 2023). Increases in PMN-MDSCs are reported as early as 24 h after experimental middle cerebral artery occlusion in mice (Kawano et al., 2019). Given that these cells suppress T cell activation and proliferation in cancer, they may play similar roles in immunosuppression after stroke.

Previous studies have reported sex differences in stroke pathology, especially relating to the immune response to stroke. Here we will highlight a few examples; however, we acknowledge this topic has previously been reviewed in depth by others (Ahnstedt and McCullough, 2019; Banerjee and McCullough, 2022; Liu et al., 2022; Tariq et al., 2023). McCullough and others found sex differences in pathways leading to cell death after middle cerebral artery occlusion (MCAO) in mice (McCullough et al., 2005). Specifically, cell death as a result of poly-ADP ribose polymerase (PARP) activation and nitric oxide toxicity is mainly restricted to males, whereas cell death for females is caspase-mediated. Another group revealed that males exhibit increased macrophages and T cells within the ischemic hemisphere compared to females 2 days after MCAO (Xiong et al., 2015). Furthermore, gene expression analysis of whole blood samples from human patients revealed females express significantly more neutrophil specific transcripts up to 3 h following cardioembolic stroke (Stamova et al., 2014). Additional research is needed to elucidate the underlying cellular mechanisms that lead to sex differences in the pathological responses to stroke, including sex differences in cells of the BBB and infiltrating immune cells. Future studies should also aim to determine the extent of hormonal and chromosomal influences that impact sex specific immune responses to stroke.

## 6 Sex differences in nanomedicine

The study of sex differences in nanomedicine is a burgeoning field. We are not the first to consider this topic (Hajipour et al., 2021; Sharifi et al., 2021; Yang et al., 2021; Poley et al., 2022). Here, we will focus on summarizing empirical observations of sex differences in the field of nanomedicine, moving from microscopic to macroscopic length scales, with a particular focus on implications for developing nanoparticle systems in acute brain injury.

### 6.1 Sex differences in nanomedicine interaction with cells and fluids

Sex differences in nanoparticle interactions with cells and biological fluids have been observed in several *in vitro* contexts. Following administration of a nanoparticle to the body, proteins that are present in biological fluids will rapidly form a coating, or corona, on the surface of the nanoparticle (Bashiri et al., 2023; Jiang et al., 2023). This protein corona is a primary driver of nanoparticle distribution and elimination, mediating both desired interactions (such as uptake of the nanoparticle by target cells) as well as undesired interactions (such as nanoparticle opsonization by circulating immune cells) (Tran and Roffler, 2023). Ashkarran and others concluded that protein corona composition was distinct for silica nanoparticles exposed to female *versus* male mouse plasma (Ashkarran et al., 2023), which may be a critical finding for the field. However, it should be noted that their results are reported from experiments involving plasma that was pooled from 3 female *versus* 3 male mice, yielding a single sample of plasma for each sex. Because nanoparticles were incubated with this pooled plasma, it is difficult to ascertain whether the reported differences could be attributed to female *versus* male sex or normal biological variability. These are important early observations that merit deeper evaluation in future work.

Data regarding how sex influences the interaction of nanoparticles with cells is unfortunately sparse. However, what has been described thus far suggests that how sex influences these interactions may depend on cell type. In one example, Serpooshan and others generated human amniotic stem cell (hAMSC) cultures from the amniotic layer of placenta from female and male fetuses (Serpooshan et al., 2018). Male cells were observed to take up fewer quantum dots than female cells in these hAMSC cultures; interestingly, this relationship was reversed in primary salivary fibroblast cells, where female cells were observed to take up fewer quantum dots than male cells. These differences in nanoparticle internalization as a function of cell sex were directly attributed to differences in arrangement and properties of actin filaments governing cytoskeletal reorganization, suggesting a cell intrinsic basis. Mahmoudi and others reported in a conference abstract that the uptake of superparamagnetic iron oxide nanoparticles (SPIONs) was threefold higher in female human inducible pluripotent stem cells (hiPSCs) compared to male hiPISCs. Similar to the work accomplished by Serpooshan, et al., these differences in nanoparticle uptake on the basis of sex were attributed to differences in actin structure and organization in female *versus* male cells (Mahmoudi, 2021). Beyond these reports, detailed analyses of NP interactions at the cellular level are otherwise lacking.

### 6.2 Sex differences in nanomedicine biodistribution in the periphery

When a nanoparticle system is developed for the treatment of disease, one of the most significant questions will be the extent to which the nanoparticle agent accumulates in different organ systems, i.e., biodistribution, since this will drive both efficacy and toxicity (Wei et al., 2018). The biodistribution of solid metal nanoparticles has received particular attention due to ubiquitous use of these materials in the human environment and concerns regarding potential long-term toxicity. Although these particular systems are not typically intended to deliver therapeutic agents, the studies that follow provide insight into the fundamental mechanisms that will govern distribution of nanomedicines. Of the metal nanoparticle family, silver nanoparticles (AgNPs) are well-studied in terms of potential sex differences, likely due the extensive use of AgNPs in textiles, cosmetics, and medical applications, such as wound healing (Islam et al., 2021). Multiple reports have observed that peripheral organ accumulation of AgNPs is higher in female subjects compared to male subjects for bare metal AgNPs, and this holds true for oral, IV, intraperitoneal (IP), subcutaneous, and inhaled routes of administration (Kim et al., 2009; Sung et al., 2009; Tariba Lovaković et al., 2021; Ćurlin et al., 2021; Mahdieh et al., 2022; Xue et al., 2023). There is modest evidence that steroidal hormones may drive these differences. Lovakovic and others administered AgNPs coated either with polyvinylpyrrodine (PVP) or the BBB-targeting ligand transferrin (TRF) to male and female mice. Mice were either left gonadally intact or received gonadectomy. Significant differences in AgNP deposition were observed in the liver for intact females *versus* intact males and in the lung for intact males *versus* gonadectomized males. Importantly, TRF-driven targeting effects were observed for the intact male group only, while TRF did not yield significant targeting for female or gonadectomized subjects. Differential inflammatory responses were observed in gonadectomized *versus* intact subjects, which supports the expectation that gonadotropins may influence distribution processes (Tariba Lovaković et al., 2021). For similar PVP-coated AgNPs administered orally to rats, nanoparticles were observed to accumulate to a much higher extent in female rats compared to male rats for blood, liver, kidney, heart, stomach, and duodenum (Ćurlin et al., 2021). In detailed work performed by Boudreau, et al., variously sized AgNPs (10, 75, 110 nm diameter) were observed to accumulate more effectively in female rats compared to male rats in the gastrointestinal tract and associated mesenteric lymph nodes (Boudreau et al., 2016). In contrast, aptamer-loaded gold nanostars were observed to accumulate in the spleens and liver of female mice at a five-fold lower level than male mice (Dam et al., 2015). These contrasting reports highlight the expectation that sex differences may be unique to the nanoparticle system under consideration.

Sex differences in nanomedicine circulation within the vascular compartment could be a likely explanation for sex-dependent deposition in peripheral organs. Boudreau and others concluded that there were no differences in the half-life of AgNPs in blood for female *versus* male subjects, however, the conclusion appears to have been drawn from semi-quantitative analysis (Boudreau et al., 2016). When we extracted the raw data from this report, there was modest evidence that the half-life of AgNPs for female mice is faster than for male mice (Supplementary Figure S1). Other studies have reported longer circulation time and slower elimination half-life for female *versus* male mice following administration of AgNPs (Xue et al., 2023). Yet other studies have reported no difference in half-life for female *versus* male mice following administration of ZnO nanoparticles (Choi et al., 2012), although it appears that Zn was primarily present in ionic rather than nanoparticle form in tissue, suggesting that organ deposition in that work was not driven by the colloidal system. Taking a broader view, traditional pharmacokinetic characterization of nanoparticle system via ADME approaches is critical; more detailed evaluation of the pharmacokinetic profiles of nanoparticles in blood of female *versus* male subjects, particularly in context of gonadectomy or other hormonal manipulations, would yield better understanding of the mechanisms driving differential organ distribution and may also guide eventual clinical dosing considerations.

### 6.3 Sex differences in the body’s response to nanomedicine

Empirical evidence suggests that female *versus* male subjects exhibit different biological and physiological responses to nanoparticle administration. Here, we will focus on observational studies that have reported sex differences in serum biochemistry and complete blood counts, oxidative stress, tissue-specific toxicity, and immune activation following parenteral administration of a nanoparticle system.

#### 6.3.1 Biochemistry and complete blood count

Whole blood is composed of both cellular (white blood cells, red blood cells) and acellular (plasma, platelets) components. Evaluation of serum biochemistry (i.e., the level of various proteins, enzymes, and lipids) or complete blood count (CBC, i.e., cellular counts and proportions) can yield insight into potential toxicity of therapeutic systems. Several recent reports suggest that biochemical and CBC responses to nanoparticle administration depend on sex (Gosselin, Ramaiah, and Earl, 2011). Lanthanum titanate nanoparticles (LT NPs) produced differing effects on both serum markers and CBC as a function of biological sex, with evidence for immunosuppression in male mice under the same conditions in which expansion of monocytes was observed in female mice (Akram et al., 2020). Male mice were also observed to respond to nanoparticle administration with increased triglyceride levels, which was not observed in females and that may raise concerns for patients at-risk for cardiovascular disease. Sex differences were observed for female *versus* male rats after treatment with CuNPs, particularly at high doses, and these differences were not observed in the free ion Cu (non-nanoparticle) groups (Riaz et al., 2020). Complex and sex-dependent effects on serum biochemistry for female *versus* male rats following administration of CuNPs have been reported by other investigators (Kim et al., 2016). Chen et al. described differences in thymus and spleen indexes for female and male mice following administration of PEGylated AuNPs, suggesting sex-dependent immunological response (Zhang et al., 2013). These differences included distinct immunological responses, as well as differences in white blood cell, red blood cell, and platelet counts that depended on dose as well as sex. In other work, male mice that received oral administration of ZnO nanoparticles showed no change in serum biochemistry, while females showed increased bilirubin compared to their control, which could suggest sex-dependent liver toxicity (Kuang et al., 2021). Sex-dependent physiological reactions to nanoparticle treatment can involve direct impact on the endocrine system; when female rats were dosed with titanium dioxide nanoparticles, subjects exhibited transient hypoglycemia on multiple days of observation, while the same dosing regimen in male rats did not cause hypoglycemia (Chen et al., 2020). Sex differences in insulin and glucagon levels likely explain these results and could be a critical sex-dependent safety consideration for individuals with aberrant glucose metabolism (e.g., in diabetes). These data support an expectation that serum biochemistry and CBC responses to nanomedicine will depend on biological sex, which is an important consideration in toxicological evaluation of nanoparticle systems.

#### 6.3.2 Oxidative stress

Oxidative stress is a major focus of CNS research in acute brain injury, being both a driver and potential therapeutic target for disease pathology (Allen, C. L. and Bayraktutan, U., 2009; Rodriguez-Rodriguez et al., 2014; Salim, 2017; Hakiminia et al., 2022). Oxidative stress involves an imbalance between reactive oxygen species (ROS) and antioxidant cellular machinery, leading to an accumulation of ROS that yield damage to intracellular lipids, proteins, and oligonucleotides. Increased oxidative stress has been linked causally to chronic inflammation and neurodegenerative processes in Alzheimer’s disease, Parkinson’s disease, amyotrophic lateral sclerosis (ALS), and multiple sclerosis (MS) (Pizzino et al., 2017). This mechanism of cellular and tissue damage is increasingly implicated in the secondary injury cascades that result from acute CNS, which is discussed in greater detail in Section 5.1.2 of this review. Early evidence suggests that oxidative responses to nanoparticle administration depend on sex, although the reported relationships are complex and are not at a stage where they can be generalized. In one study, researchers studied biocompatibility of low- and high-dose (LD and HD, respectively) PVP-coated AgNPs (Ćurlin et al., 2021). Females and males displayed distinct patterns of peroxyl radical accumulation that depended both on the particle type as well as the organ system examined. Interestingly, female liver and kidneys both showed a decrease in superoxide dismutase (SOD) levels for the LD group and a return to control values in the HD group. In contrast, levels of SOD in male rats did not show a clear pattern between liver and kidney or between HD and LD. In separate work utilizing a nanoparticle with specific intent of reducing catalase and SOD, significant reduction of oxidative stress in male mice was achieved with lower ratios of SOD1:CAT in nanoparticles, whereas the ratio was doubled in females to achieve the same effect (Tarudji et al., 2023). Complex antioxidant responses were observed to also depend on sex in specific organ systems, including heart, liver, and kidney for lanthium titanate NPs (Akram et al., 2020). Treatment with AgNPs nanoparticles yielded significant changes in oxidative stress markers, such as peroxyl radicals and superoxide radicals, in male and female mouse kidneys, livers, brains and lungs. Differences were not only observed between male and female mice, but there were also differences based on whether the mice were intact or gonadectomized, which highlights the important role that gonadotropin signaling may play (Tariba Lovaković et al., 2021). Taken in sum, these data suggest that oxidative stress responses can be sex-specific, and that these differences should be considered for the development of new therapeutics.

#### 6.3.3 Inflammation and toxicity

Growing evidence suggests that sex differences play a role in driving phenotypic, organ-level, or cellular toxicity. In one recent and high profile example, major differences have been observed for tolerability of the solid lipid nanoparticle COVID-19 vaccine, with females reporting significantly higher adverse effects immediately following inoculation and males experience higher susceptibility to adverse cardiac events at later time points (Duijster et al., 2023); the fact that the vaccine is reported to induce cycle irregularities in menstruating individuals (Rahimi Mansour et al., 2023) suggests that endocrine or gonadotropic signaling may be involved. We note that, given the recency of these observations, sex differences in COVID-19 vaccine tolerability is an area of active clinical investigation that is evolving rapidly. Preclinically, sex differences in toxicity have been studied in various formats. Sex differences were observed for inhaled Titanium Dioxide nanoparticles (TiO_2_-NPs), such that male rats exposed to TiO_2_-NPs exhibited a significant increase in circulating neutrophils, while female rats were apparently more susceptible to toxicity at the organ level (Yamano et al., 2022). Phenotypic evidence of toxicity was evident as pulmonary dust foci (PDF), which presented as milky white spots on the lungs and were proposed to be agglomerations of macrophages; PDFs were seen in females at lower NP doses compared to males, which suggests that females may be more sensitive to the toxicities related to TiO_2_ inhalation. Similarly, female rats were observed to be more susceptible to hepatic toxicity, with increased levels of biomarkers for oxidative stress after oral administration. In contrast to these results, a different study observed that females were less susceptible to lung toxicity than males following exposure to ZnO-NPs (Sehsah et al., 2022); although the initial experiments would have suggested a simple or generalizable dependence of toxicity on sex, their deeper analyses that examined genes regulating oxidative stress responses and inflammation-associated chemokines revealed highly complex regulation of individual pathways. Yang et al. demonstrated that there were significant changes in hormone production in the female rats treated with copper nanoparticles (CuNPs), as well as heightened expression of caspase proteases following parenteral administration in females, although they did not compare these results to male mice (Yang et al., 2017). Other research has also shown an increase in micronucleation of immune cells, with a higher quantity of these cells in male *versus* female rats across multiple dosing groups by the oral route (Kim, Soon Yong et al., 2008). In detailed studies performed by Han and others, amorphous silica nanoparticles (ASiNPs) instilled intratracheally yielded the highest level of lung damage in female rats compared to males (Han et al., 2020). Expression of caveolin-1 and matrix metalloproteases in females were suggested to account for these differences. In separate studies, cytokine marker expression levels depended on sex. Gokulan and others reported that a T-cell marker was significantly increased in male tissues after 24 h, but the same marker was significantly decreased in females at the same time point and dose (Gokulan et al., 2020). Taken in sum, these data support an expectation that toxicity of nanoparticle systems will depend on sex, although clearly these relationships are complex and may be unique to each nanoparticle systems.

### 6.4 Sex differences in nanoparticle delivery to the injured brain

Nanoparticle delivery systems provide injectable, sustained release options of therapeutic intervention for various diseases and injuries. For neural applications, the largest obstacle to consider when designing drug delivery vehicles is the BBB, which larger sized particles (i.e., >20 nm) are typically unable to cross (Cook et al., 2015; Medina et al., 2017). However, the breakdown of the BBB after injury gives rise to unique opportunities to use nanoparticle drug carriers. Drug loaded nanoparticles can be tailored to suit a desired therapeutic target, which may include reducing oxidative stress, protecting against cellular apoptosis, or modulation of the immune system. While nanoparticle-based drug delivery systems for brain injury are not currently available in the clinic, these systems are clinically approved for the treatment of various diseases including cancer, hemophilia, and multiple sclerosis (Anselmo and Mitragotri, 2019; Mitchell et al., 2021). The following paragraphs discuss the preclinical reports of sex differences in systemic nanoparticle delivery to the injured brain.

Sex differences have been observed in the development of treatments for TBI. Our group has previously investigated nanoparticle accumulation profiles in male and female cerebral cortex after experimental TBI (Bharadwaj et al., 2020). Fluorescence microscopy confirmed greater nanoparticle accumulation at 24 h post-injury in the female injured cortex compared to the male injured cortex. This study also revealed sex differences in the temporal nanoparticle accumulation profile spanning up to 7 days after injury. Males exhibit a biphasic accumulation pattern with greater accumulation at 3 h and 3 days compared to 1 day after injury, however females do not share this same temporal profile. This finding is indicative of sex-dependent BBB dysfunction and is the focus of ongoing studies. Tarudji et al. reports the overall contrast extravasation rate of antioxidant enzyme loaded PLGA nanoparticles was significantly lower in female mice than male mice at 24 h after experimental TBI (Tarudji et al., 2023). Although these studies used the same injury model, this opposing finding may be due to differences in injury severity parameters, given that Bharadwaj et al. employed a 2 mm depth impact and Tarudji et al. employed a 2.5 mm impact. Previous studies characterizing the CCI model have shown that injury severity is dependent on impact depth, speed and diameter, with more severe injuries resulting from increases in these parameters (Saatman et al., 2006; Osier and Dixon, 2016). Together, these studies show that there are sex differences in nanoparticle delivery to the injured brain. Further research is needed to investigate the underlying mechanisms that cause sex-dependent pathologies seen in animal models of TBI and how these mechanisms can be harnessed to improve therapeutic delivery. Additionally, the type of injury model (cortical controlled impact, fluid percussion injury, blast-induced injury) and severity of the injury may impact the magnitude and temporal trajectory of sex differences and should be considered carefully in future research.

Similar to TBI, the dependence of efficacy on sex has been observed in preclinical studies for stroke. For example, Challa et al. explored the impact of MMP-12 slicing after ischemic stroke by delivering MMP-12 shRNA loaded nanoemulsions IV to male and female mice at different time points post-reperfusion (Challa et al., 2022). MMP-12 is a known player in amplifying local and systemic immune response as well as prolonging BBB disruption. Animals receiving the MMP-12 shRNA exhibited a significant knockdown of MMP-12 within the ipsilateral hemisphere compared to control plasmids and sham animal controls. When neuromotor behavior was assessed, a sex-dependent effect was observed with MMP-12 knockdown therapy. Specifically, a higher level of therapeutic impact for neurological motor behavior was observed in male rats compared to female rats (Challa et al., 2022). It should be noted that the stroke induced motor behavior deficit was not as significant in female rats compared to male rats (Koellhoffer and McCullough, 2013). This phenomenon is well-documented in the preclinical stroke research field, where it is hypothesized that circulating sex hormones may play a role in the molecular sequelae and thus the functional deficits that emerge. Other studies have demonstrated sex-dependent response to small molecule or antibody therapeutics, however, the PD/PK for nanoparticle systems in preclinical stroke models have yet to be fully explored and characterized (Ruddy et al., 2019; Seifert et al., 2019; Tejeda-Bayron et al., 2021). This area of research is ripe for further assessment.

## 7 Conclusion/call to action

The main objective of this review was to highlight the current knowledge of if and how sex-dependent biological variables impact nanomedicine within the context of acute brain injury pathologies. We can conclude from our analyses that sex is an important consideration. We also identify critical gaps and challenges for the field. This includes a lack of consideration of cell sex in *in vitro* studies, oversimplification or failure to track estrus cycles in experimental design, lack of balanced experimental designs powered for detecting sex differences, and a lack of foundational work directly evaluating nanomedicine-specific mechanisms that may drive sex differences (for example, differences in protein corona driving differences in circulation time).

We suggest the following call to action:

- Train all scientists in the distinction between gender and sex as well as the complexity of physiological factors that drive sex differences
- Elevate field-standard expectations that published work will consistently and directly provide rationale regarding
  - Inclusion or exclusion of sex as a biological variable in all experimental work
  - Inclusion or exclusion of estrus cycle tracking in preclinical experimental designs
- Deepen mechanistic rigor for nanomedicine development to account difference in sex-dependent microenvironmental factors through
  - Increased attention to foundational aspects of protein corona formation and nanoparticle distribution and clearance as a function of sex
  - Standard inclusion and description of cell sex for *in vitro* experiments
  - Utilizing preclinical manipulations such as hormone replacement, gonadectomy, or transgenic or chimeric models to better understand the mechanistic basis for sex differences
- Develop and support team science initiates that would facilitate the collaboration of diverse scientists and thought leaders capable of pursuing this complex, cross-disciplinary work

We further challenge the field to consider the real-world context for development of new nanomedicines, including addressing sex as a biological variable in clinical populations that are atypical in their sex identity or hormonal profiles as a result of typical aging processes, hormone replacement therapy, intersex conditions, non-binary gender presentation, or gender dysphoria.

Here, we address the intersection of nanomedicine and acute brain injury in the context of biological sex. This field is poised to yield new therapeutic approaches and mechanistic understanding that will positively impact human health. We are encouraged by growing recognition and incorporation of sex as a biological variable and look forward to clinical advancements in the years to come.

## Supplementary Material

Supplemental data
