# Supplementary material for: Acute brain injury and nanomedicine: sex as a biological variable
Source: Front Biomater Sci. Author manuscript; Available in PMC 2024 Oct 24. (PMC11500709; doi:10.3389/fbiom.2024.1348165)
Supplement: Supplemental data [file NIHMS2029528-supplement-Supplemental_data.pdf]

Supplementary Information

Figure 2. Percent difference was calculated by the following formula: (male-female)/female. Values on the left side of the x-axis thus represent parameters that are higher in female subjects, while values on the right side of the x-axis represent parameters that are higher in male subjects.

Detailed sources and raw data follow:

Lean body mass (kg), Body fat percentage (%), Whole heart mass (g), Body mass (kg), Body surface area (m^2), Heart rate (bpm), Cardiac output (L/min), aortic systolic blood pressure (mmHg), and aortic diastolic blood pressure (mmHg) values were obtained from published work (St. Pierre *et al*, 2022).

White blood cell counts ($x10^3$ uL), red blood cell counts ($x\ 10^6$ uL), hemoglobin (g/dl), hematocrit (%), and platelet count ($x10^3$ uL) values were obtained from reference ranges compiled by the Centers for Disease Control.

Grey matter mean cerebral blood flow (ml/100g/min) and white matter mean cerebral blood flow (ml/100g/min) values were obtained from published work (Alish *et al*, 2021).

Gastric pH and gastric emptying time (min) values were obtained from published work (Soldin and Mattison, 2009, and Mori *et al*, 2017, respectively).

**Parameter**	**% diff**
Body fat percentage (%)	-44.92
Grey matter mean CBF (ml/100g/min)	-32.61
Gastric pH	-25.87
gastric emptying time (min)	-20.34
White matter mean CBF (ml/100g/min)	-10.53
platelets (x10^3 uL)	-8.64
Heart rate (bpm)	-6.07
white blood cell (x10^3 uL)	-2.44
Aortic diastolic blood pressure (mmHg)	6.35
Aortic systolic blood pressure (mmHg)	9.36
red blood cell (x 10^6 uL)	11.24
hematocrit (%)	14.68
hemoglobin (g/dl)	16.41
Body surface area (m2)	17.18
Body mass (kg)	25.64
Cardiac output (L/min)	28.26
Whole heart mass (g)	35.10
Lean body mass (kg)	55.34

Alisch, J.S.R. *et al.* (2021) ‘Sex and age-related differences in cerebral blood flow investigated using pseudo-continuous arterial spin labeling magnetic resonance imaging’, *Aging (Albany, NY)*, 13(4), pp. 4911-4925. Available at: https://doi.org/10.18632/aging.202673

Mori, H *et al.* (1961) ‘Gender Difference of Gastric Emptying in Healthy Volunteers and Patients with Functional Dyspepsia’, *Digestion*, 95(1), pp. 72-78. Available at: https://doi.org/10.1159/000452359

Soldin, O.P., and Mattison, D. R. (2009) ‘Sex differences in pharmacokinetics and pharmacodynamics’, *Clinical Pharmacokinetics*, 48(3), pp. 143-157. Available at: https://doi.org/10.2165/00003088-200948030-00001

St. Pierre, S.R. *et al.* (2022) ‘Sex Matters: A Comprehensive Comparison of Female and Male Hearts’, *Frontiers in Physiology*, 13(831179). Available at: https://doi.org/10.3389/fphys.2022.831179

United States, Department of Health and Human Resources, Centers for Disease Control and Prevention. ‘Complete Blood Count (CBC) with Five-Part Differential’ *Centers for Disease Control and Prevention*, https://www.cdc.gov/nchs/data/nhanes/nhanes_03_04/l25_c_met_complete_blood_count.pdf (accessed on Nov 28th, 2023)

Supplementary Figure

	AgAc - F	10nm - F	75nm - F	110nm - F	AgAc - M	10nm - M	75nm - M	110nm - M
Elim half-life (hours)	14.10	11.07	12.13	19.46	28.64	16.47	26.63	59.68

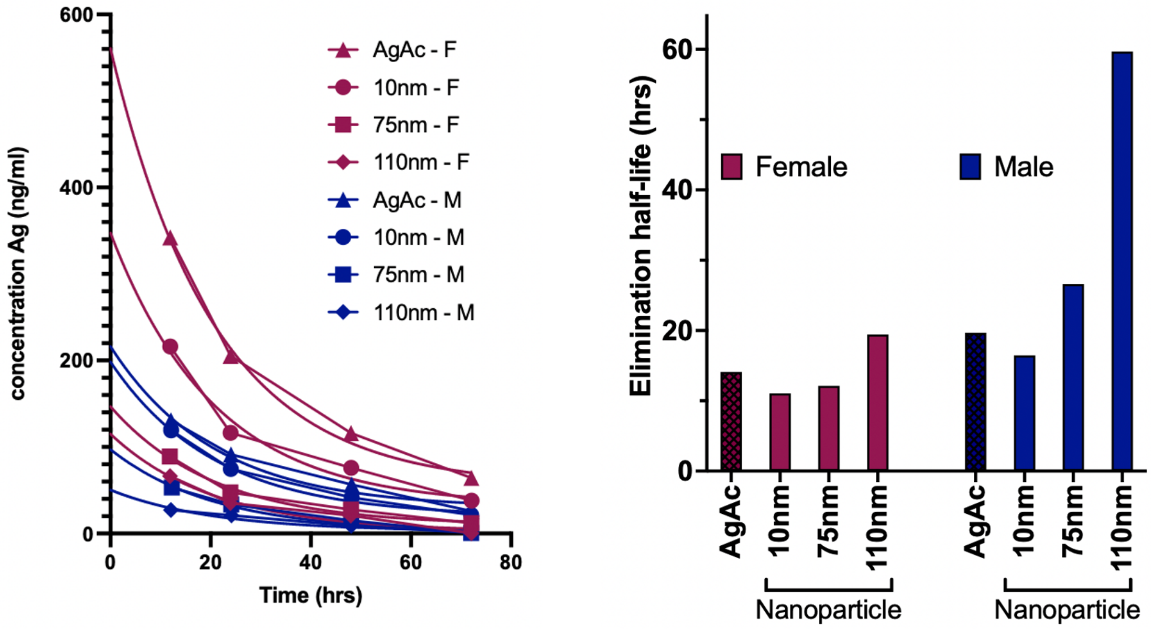

Supplementary Figure 1. The elimination half-life of AgNPs may depend on sex. Pharmacokinetic values of Ag concentration in plasma were extracted from published work (Boudreau *et al*, 2016). In these studies, rats received either AgOAc (the ionic form of silver) or AgNPs (the nanoparticle form of silver) by oral gavage, and plasma samples were collected via tail vein. Although the elimination half-life appears to be generally similar for female versus male rats that received ionic silver, the elimination half life appears to be smaller for female versus male rats that received silver nanoparticles, with some evidence that the effect is greater for larger diameter nanoparticles.

<u>Methods</u>

Data were extracted from Figures 3A and 3B (Boudreau *et al,* 2016). A web-based tool (PlotDigitizer, PORBITAL, https://plotdigitizer.com) was used for data extraction. Extraction was accomplished by calibrating the X-Y scale to known values and manually positioning marks over each data point with a curser. The data were analyzed in Graphpad Prism (version 10), and simple exponential decay curve fits were applied to the elimination phase only (12 hr time point).

Boudreau, M.D. *et al.* (2016) 'Differential Effects of Silver Nanoparticles and Silver Ions on Tissue Accumulation, Distribution, and Toxicity in the Sprague Dawley Rat Following Daily Oral Gavage Administration for 13 Weeks', *Toxicological Sciences*, 150(1), pp. 131–160. Available at: https://doi.org/10.1093/toxsci/kfv318.
